# Training for Staff of an Employment Training Program to Promote Mental Health Discussions and Referrals With Out-of-School Youth, Baltimore, Maryland, 2007

**Published:** 2012-01-26

**Authors:** Margaret Gifford Tucker, S. Darius Tandon, Freya Sonenstein

**Affiliations:** Johns Hopkins University Bloomberg School of Public Health; Johns Hopkins University School of Medicine; Baltimore, Maryland; Johns Hopkins University School of Medicine; Baltimore, Maryland

## Abstract

We examined whether mental health training for staff of an employment training program for out-of-school youth aged 16 to 22 years would increase mental health discussions and referrals. We reviewed case files of participants at 1 Baltimore program who enrolled 6 months before (n = 303) and after (n = 263) a 2-day training program. Chi-square analyses indicated increases in the percentage of participants with discussions (1% to 9%, χ^2^ = 4.91, *P* < .05) and referrals (11% to 16%, χ^2^ = 5.16, *P* < .05). Brief, intensive training increased mental health discussion and referrals among job training staff.

## Objective

We examined whether mental health training for staff in a youth employment training program would result in increased mental health discussions and referrals by staff with program participants. Out-of-school youth often turn to such programs staffed by employees who manage participants' employment and education goals (1). These programs typically serve residents of high-poverty neighborhoods with increased risk for depression, substance abuse, and trauma-related disorders (2-5). Training staff to identify mental health risks may increase awareness and access to services in this population. We know of no studies examining the effect of mental health training of employment training staff.

## Methods

We conducted an observational case file review of 2 cohorts of participants enrolled at an employment training program serving predominantly low-income, African American youth aged 16 to 22 years. Participants in this program were out-of-school youth who have a high school diploma or who dropped out of high school and were looking for employment training. The Johns Hopkins University institutional review board approved this study. This program is described in detail elsewhere (5).

Cohort A participants were enrolled 6 months before training, and cohort B participants were enrolled in the 6 months after the training. The 2-day training was provided to all 12 staff at the Baltimore Youth Opportunity (YO) Center to emphasize the importance of mental health discussion (ie, on-site discussion of a life event or issue relating to mental health) and referral to mental health services for participants with poor mental health. A description of topics covered is in Table 1.

The review period for each cohort was limited to pretraining and posttraining to ensure that the files from members in the pretraining cohort were not reviewed after the staff training. A structured abstraction form and operational definitions (Table 2) for each variable to be abstracted were created. Three trained research assistants used these forms to document each interaction between staff and participants during each 6-month cohort. For each interaction, the research assistant documented whether mental health-related discussions or referrals to on- or off-site mental health services were noted. The first and second authors reviewed the first 25 case files to ensure reliability and met with research assistants on a biweekly basis to resolve questions about the abstraction process. Eight of the 12 staff trained were case advocates who had direct contact with participants and documented discussions and referrals; there were no changes in the YO staff between the first and second cohort periods.

Data were analyzed by using Stata version 10.0 (StataCorp LP, College Station, Texas). Chi-square analyses were used to calculate differences between the 2 cohorts in the percentage of interactions during which mental health discussions and referrals took place.

## Results

A total of 303 files were reviewed for participants enrolled before the staff training (cohort A) and 263 files were reviewed for participants enrolled posttraining (cohort B). The average number of visits for each participant with a case advocate during the 6-month period of review was 7 for cohort A and 5 for cohort B. Across groups, program participants were predominantly African American (99%), 50% male, and were aged 19 years on average.

In the posttraining cohort, 9.3% of participants had a mental health discussion with staff compared with 1.3% among the cohort measured before staff training (χ^2^ = 4.91, *P* < .05). Additionally, the percentage of participants who had a mental health referral was higher after the training (19%) than before the training (15.9% vs 11.3%, χ^2^ = 5.16, *P* < .05).

## Discussion

We examined whether staff training would affect the number of mental health discussions and referrals at an employment training program for youth. Referrals and mental health discussions increased in the 6 months after the training, which may reflect the training's focus on identification of poor mental health, awareness of mental health services available, and awareness of boundaries regarding appropriate mental health discussion for staff to have with a program participant. A higher percentage of discussions may be due, in part, to a greater awareness among staff of how the challenges commonly faced by their clients can trigger symptoms of poor mental health, such as community violence triggering anxiety or posttraumatic stress disorder. The increase in referrals after the training may be attributable to the emphasis the training placed on boundaries for discussing mental health-related issues during interactions. Referral to the on-site mental health clinician was emphasized as an option.

Our findings are subject to 3 limitations. First, data on discussions and referrals are dependent on staff documenting interactions in case files. Second, the cohort review ended after 6 months; therefore, some participants in each cohort may have had discussions or referrals after the 6-month period that have been missed during data abstraction. Finally, this study examined limited outcomes and may have benefited from additional evaluation.

Our findings suggest that training staff in employment training programs may increase the number of mental health referrals and discussions in a population that may otherwise not receive appropriate treatment. Training of staff was brief and inexpensive, yet it increased mental health discussion and referral. Such trainings could be integrated into various types of programs specializing in developing competencies that do not focus on health issues. These findings demonstrate the benefits of providing mental health training to staff in programs serving out-of-school youth.

## Figures and Tables

**Table 1.** Mental Health Topics Covered at 2-Day Training^a^ for Paraprofessional Staff in Baltimore, Maryland, March 1-2, 2007

**Topic**	Topic Details
General description of mental health	Adolescent and young adult depression and anxiety disorders: prevalence, symptoms, sequelae.
Substance abuse	Substance use disorders, drug and alcohol abuse, self-medication, medication use while in subtreatment, relationship to mental health.
Violence	Trauma and posttraumatic stress disorder definitions and symptoms, violence among teens, violence as a public health issue.
Adolescent brain development	Prefrontal cortex development, emotion and reason, judgment, emergence of risk-taking behaviors.
Mental health and the law	Confidentiality, records and proper documentation, consent, legally required disclosure.
Staff responsibilities in addressing mental health	Staff roles, approaches for referring participants to on- and off-site mental health services, fostering relationships with participants to discuss mental health issues; discussion of boundaries among staff when discussing mental health with participants (eg, caution in not diagnosing, caution in approaching topics more safely handled by trained professionals).

a The training was a compilation of evidence-based curricula developed by Johns Hopkins faculty and guest clinical speakers from local mental health agencies and is available from the authors.

**Table 2.** Operational Definitions for Data Abstraction

**Variable**	Operational Definition
Cohort	Cohort A: program participants with enrollment dates of September 1, 2006 through February 28, 2007. Cohort B: program participants with enrollment dates of March 1, 2007 through August 31, 2007.
Discussion of mental health	Any notes in case file that indicate the following: Case advocate talked to member about mental health. Mental health includes, but is not limited to, topics of depression, anxiety, anger management, aggressive behavior, stress, and exposure to violence. Discussion of mental health does not include asking client in general terms how he or she is feeling. OR Case advocate gave information (not referral) to member about mental health.
Mental health topics discussed	List specific mental health topic(s) that the case advocate discussed with YO member.
Referral to YO counselor given to YO member	Notation in case file that YO member was referred to the YO counselor for mental health related reason.
Referral to outside mental health resource given to YO member	Notation in case file that YO member was referred to an outside mental health resource.

Abbreviation: YO, Baltimore Youth Opportunity Center.
